# The Release of Trace Elements in the Process of Coal Coking

**DOI:** 10.1100/2012/294927

**Published:** 2011-12-18

**Authors:** Jan Konieczyński, Elwira Zajusz-Zubek, Magdalena Jabłońska

**Affiliations:** ^1^Institute of Environmental Engineering, Polish Academy of Sciences, 34 M. Skłodowskiej-Curie Street, 41-819 Zabrze, Poland; ^2^Department of Air Protection, Silesian University of Technology, 2 Akademicka Street, 44-100 Gliwice, Poland

## Abstract

In order to assess the penetration of individual trace elements into the air through their release in the coal coking process, it is necessary to determine the loss of these elements by comparing their contents in the charge coal and in coke obtained. The present research covered four coke oven batteries differing in age, technology, and technical equipment. By using mercury analyzer MA-2 and the method of ICP MS As, Be, Cd, Co, Hg, Mn, Ni, Se, Sr, Tl, V, and Zn were determined in samples of charge coal and yielded coke. Basing on the analyses results, the release coefficients of selected elements were determined. Their values ranged from 0.5 to 94%. High volatility of cadmium, mercury, and thallium was confirmed. The tests have shown that although the results refer to the selected case studies, it may be concluded that the air purity is affected by controlled emission occurring when coke oven batteries are fired by crude coke oven gas. Fugitive emission of the trace elements investigated, occurring due to coke oven leaks and openings, is small and, is not a real threat to the environment except mercury.

## 1. Introduction

In recent years, a growing interest in the impact of micropollutants, such as heavy metals, on the environment pollution and human organism has been observed. More than a dozen trace elements, including mercury, are considered harmful to humans, and therefore their emissions should be eliminated or significantly reduced. The threat, taking into account the toxicity, refers to 12 elements: As, Be, Cl, Cr, Cd, Co, Hg, Mn, Ni, Pb, Sb, and Se (Hazardous Air Pollutants (HAPs), US EPA) [1].

Essential part of the hazardous elements emission results from the coal processing and its use as energy source. Hazardous elements, occurring in coal in trace amounts, are emitted with the flue gases from furnaces or exhaust gases from the processing operations. They may also move to other parts of the environment through the processing products of or combustion byproducts like ash, slag, and gypsum from flue gas desulphurization.

Pyrolysis of coal used in coke industry leads to the release of hazardous elements together with the volatile products and their distribution between coke, gas and liquid products, and streams of volatile contaminants emitted from the coke oven battery [2]. Therefore, researches have been conducted on the health hazards of people living in the regions affected by coking plants activity. Heavy metals, besides being an environmental problem at the local level, are also a serious problem on a continental scale and in the case of mercury, even on a global scale. The issue is serious owing to the fact that the world production of coke is still growing and will soon reach 800 million Mg/year.

Knowledge of the behaviour and assessment of the emission of trace elements in a coke production process will contribute to a better understanding of the environmental threat and will yield more precise data on global emissions of the substances that threaten human health.

## 2. Trace Elements in the Coking Coal Process

Research on the formation of pollutants during the technological process, distribution of trace elements in solid and gaseous products of coal pyrolysis, and assessment of the emission size have been carried out by many authors.

The studies of trace elements behaviour in coal coking process focused on the differences in the amount of released trace elements depending on the way they were bound to the organic coal substance, the coal type and the process conditions, particularly the temperature of the pyrolysis process. The relationship between inorganic substance and the kinetics of coal pyrolysis was studied to a lesser extent [3–9]. Jakowlewa and Doujanskaja [2] showed that trace elements are different in the release dynamics during a coal pyrolysis process: Co, Mn, Ni, Sr, are released evenly, dynamics of As release increases whereas Cd and Cu dynamics decreases. The study of environmental hazards of certain trace elements in a coking plant and in its neighbourhood, carried out in German coking plants by Eisenhut [10], yielded consistent mass balances of Co, Cr, Cu, Ni, Pb, and V in coal and coking products. Having examined the trace element concentrations in workplaces in the coking plant, he found them not to be harmful for the employees. He also defined the levels of 21 trace elements contents in particulate matter at the distance of 300 m from two coke oven batteries. The values exceeding German standards were recorded only for 7 of the tested elements, namely, Cd, Cr, Cu, Mn, Pb, Sn, and Zn. In case of other elements determined, that is, As, Sb, Be, Co, Ni, Hg, Pd, Se, Ag, Tl, Te, V, Rh, and Pt, their contents in the air were less than the acceptable standard values. Boyd [9] studied the behaviour of trace elements in the coking process of Australian coals by analyzing samples of charge coal blends and coke. Behaviour of trace elements is related to the forms of individual elements occurrence in coal and volatility of particular element. It was found that most of the analyzed elements, except of the most volatile ones, were retained in the coke. Studies prior to [3], carried out by the same authors, covered Upper Silesian coking coal pyrolysis. It was found that the release rates varied from the highest for Hg and Cd, average for Se, and the lowest for Ni, Mn, and As. There are also new reports of the issue. Li et al. [4, 5] studied volatility of some trace elements in Chinese coals during pyrolysis in a reactor heated in the temperature ranges 950–1300°C and 950–1400°C. Research on the influence of temperature and heating rate on the release of trace elements showed that Cd, Se, and As were the most volatile elements, whereas volatility of Zn, Sr, and Ni was average. When increasing pyrolysis temperature, an increase in the release dynamics was observed for most elements studied. Having tested the behaviour of 44 elements in Chinese coal pyrolysis in the laboratory. Yiwei et al. [6] confirmed that the release dynamics increased with pyrolysis temperature, and the volatility of the elements (vulnerability to the release) increased according to the following order: rare earth metals, heavy metals, and light elements-non-metals. To explain the behaviour of some selected hazardous elements (V, Cr, Mn, Co, Ni, and Cu) during coal pyrolysis, the same authors [7] studied the forms of occurrence of these elements in coal using a sequential extraction with different solvents. They found that most of the analyzed elements in the coal occurred in organic matter of coal and extraction residue composed of silicates and sulphides. They also confirmed their earlier findings that the release rate of the tested elements increased with pyrolysis temperature and that the release rate of heavy metals was greater than the release rate of rare earth metals. By examining the forms of As and their transformation during pyrolysis of Chinese coals [8], using speciation analysis, it was found that about 72% of As in these coals are form sulfide compounds, 16% form sulphate, phosphate, and oxide compounds, 10% are As organic forms, and 2% are aluminosilicates. Organic forms of As are the most volatile, while As present in the form of aluminosilicates is of the lowest volatility.

Though the level of harmful elements emission in a coal coking process is not sufficiently well recognized, emission factors of some trace elements in a coking plant were determined on the basis of the available data [11, 12]. They range (in g/t of coke) from 0.01 for As, Hg, and Cd; 0.1 for Cu and Ni; 0.15 for Cr; 0.25 for Pb up to 0.4 for Zn [13]. Further data [14] define the following ranges of emission factor values in g/t of coke: from 0.01 to 0.03 for Hg, from 0.02 to 0.04 for Cd, from 0.08 to 0.2 for As, from 0.2 to 0.3 for Ni, and from 0.6 to 1.7 for Pb [15]. There are few reports [16–18] on the occurrence of trace elements both in coal coking products and sewage.

## 3. Research Concept and Scope

In order to assess penetration of trace elements released into the air in the process of coal coking, it is necessary to determine the loss of these elements by comparing their contents in the industrial charge coal blends and in the coke obtained from them in coke oven batteries. Knowing the value of the release coefficient and the emission factor of a given element, it is possible to calculate the emission magnitude. The present research has focused on As, Be, Cd, Co, Hg, Mn, Ni, Se, Sr, Tl, V, and Zn found in coal and coke obtained from it. Taking into account the impact of technical and technological conditions of coke production on the release of trace elements, four Polish coke oven batteries, differing in age, technology, and technical equipment, were selected for the tests. In result of the test, preliminary information will be acquired, necessary to identify the scale of trace elements emission and to evaluate any potential risks of these substances emission in a coking process.

## 4. Characteristics of the Tested Coke Oven Batteries

### 4.1. Coking Plant Victoria

The otto system battery, constructed in 1985, contains 35 coking chambers of the dimensions (m) 13,03x3,65x0,45. The chambers are stamp charged. The product is foundry coke. The daily production of coke is 1400 Mg, and the annual production is about 500000 Mg.

### 4.2. Coking Plant Carbo-Koks

The Otto system battery, modernized in 2001, contains 45 coking chambers of the dimensions (m) 13,03x3,65x0,45. The chambers are stamp charged. The daily coke production is 360 Mg, and the annual production reaches approximately 130000 Mg.

### 4.3. Coking Plant Dębieńsko

Coke oven battery, constructed in 1985, contains two blocks of 28 coking chambers of the dimensions (m) 12,30x3,80x0,47. The chambers are top charged. The daily production of coke is 690 Mg, and the annual production reaches approximately 250000 Mg.

### 4.4. Coking Plant Przyjaźń

Coke oven battery No. 5, put into use in 2007, contains two blocks of 38 coking chambers of the dimensions (m) 15,04x5,50x0,41. The chambers are top charged. In order to limit the emission of contaminants when coke is pushed out, a hood fitted with an exhaust and dust removal installation was installed above the coke guide. There are two stages of the exhaust gases dedusting: a cyclone and a bag filter. The daily production of coke is 1900 Mg, and the annual production is about 750000 Mg.

## 5. Research Methodology

### 5.1. Technical Analysis

Ash contents in the samples of coals and cokes were determined by thermogravimetric and gravimetric method in accordance with Polish standard PN-80G-04512. Moisture in coal and coke was determined using Polish standard PN-80G-04511.Volatiles in the coal samples were determined according to the Polish standard PN-81G-04516.

Total sulphur in coal was determined according to Method B of ASTM-D 4239-05 standard. In order to determine total sulphur in cokes, ASTM-D 4239-05 Method B was used. All the determinations were performed within the accreditation AB 300. Three parallel samples were analyzed.

### 5.2. Elemental Analysis

Elemental analysis (C, H, and N) was performed using a Perkin Elmer Analyzer 2400 Series II CHNS/O. The determination error was 0.2% for C and 0.1% for H and N. Three parallel samples were analyzed.

### 5.3. Determination of Mercury in Coal and Coke

Mercury contents in coal and coke samples was determined by MA-2 Nippon Instruments Corporation mercury analyzer. The method involves thermal decomposition of a sample. Three parallel samples were analysed. Atomic absorption spectrometry-cold vapour technique (CVAAS) was used to measure the mercury contents in solid analytical samples. The determination error was 0.002 ng. The accuracy of the mercury contents determination was verified using Standard Reference Material 1632c: Trace Elements in Coal (bituminous): US Department of Commerce: National Institute of Standards and Technology, Gaithersburg, MD 20899.

### 5.4. Determination of Other Trace Elements in Coal and Coke

Solid samples weighing 0.15 g were mineralized under high pressure (maximum pressure 60 bar) and high temperature (maximum temperature 260°C) in a system for microwave mineralization (Anton Paar Multiwave 3000) in an appropriate mixture of acids, which allowed to obtain a clear solution. Three parallel samples were analysed. Mineralization was carried out in two stages. In the first stage, the mixture of nitric acid, perchloric acid, and hydrofluoric acid was used. In the second stage, boric acid was added to the reaction vessel. Pure acids for trace analysis from Sigma Aldrich TraceSELECT were used. Having evaluated the amount of the analyte introduced into the sample during the process of mineralization with the mixture of the acids used, it was found that this amount did not exceed 1.5% of the concentration of the analytes in the sample. The analyses of the mineralized samples were carried out by ICP-MS method using Perkin-Elmer Sciex Elan 6100 DRC. The system was equipped with an Cetax 500 autosampler and a crossflow nebulizer. The spectrometer was optimized to provide maximal intensity for ^24 ^Mg, ^115^In, ^238^U, and minimal values for CeO/Ce (below 3%) and Ba^2+^/Ba (below 3%).

The optimum measurement conditions are presented in Table 1. The detection and quantification limits of analyzed elements are shown in Table 2. Selenium was measured using DRC-e conditions. Before analysis, all samples were diluted 1 : 10 with high purity water. Each mineralized sample was analyzed three times. All reagents used for titration were analytically pure. High purity double-distilled and deionized water for dilution and all titration reagents preparation were obtained using a Millipore's Milli-Q system. All solutions of multielemental (Merck, Germany) and monoelemental ICP-MS standards were prepared daily by the dissolution reference materials in water obtained from Milli-Q system and used for the calibration. Standards, blanks, and samples were measured using ^103^Rh as internal standard (10 *μ*g/L, Merck, Germany). Solution of 10 *μ*g/L Rh was moved into all solutions on line, by second tubing on peristaltic pomp. The uncertainties of the determination were different for different elements (As-15%, Be-17%, Cd-6%, Mn-5%, Co-5%, Ni-12%, Se-15%, Sr-7%, Tl-15%, V-7%, and Zn-17%).

## 6. Tests Results and Discussion of the Results

### 6.1. Industrial and Laboratory Tests

The samples of the charge coal and the coke obtained from them were subjected to technical analysis, and moisture, ash, total sulphur, and volatiles were determined. Elemental analyses were also carried out, and carbon C, hydrogen H, and oxygen O were determined. The results can be considered typical for coal and coke. These results are shown in Table 3.

Table 4 presents the results of determination of the tested trace elements concentrations in the samples of charge coal and coke obtained from them. Concentrations of individual elements vary considerably. The lowest values, from a few to more than 100 ppb, are for Cd and Hg. The highest values, exceeding 100 ppm and 200 ppm, are Mn and Sr concentrations, respectively. Ni, V, and Zn are present at concentrations of tens of ppm. Be, Se, and Tl are present at concentrations from a fraction of ppm to more than 1 ppm, and Co—at concentration of a few ppm.

When comparing the contents of trace elements in four charge coal, one can find almost equalized level of Hg, about 200% differences in the case of Be, Co, Mn, Ni, Sr, V, and Zn levels, differences of about 300% for Se, 400% for Tl, and 600% for As and Cd. This is obvious owing to the different levels of trace elements contents in coals, depending on the coal type, origin, basin, and other factors.

In order to evaluate the release of the trace elements in the process of coal coking, basing on the results of determination of concentration of *i* element in a *c*_icoal_ charge coal sample and in *c*_icoke_ sample of the coke obtained, a release coefficient *R*_*i*_ was derived after the following equation:

(1) $$R_{i}\;=\frac{C_{\text{icoal}}-C_{\text{icoke}}}{C_{\text{icoal}}}\cdot 100\left[\text{\%}\right],$$

where, *C*_icoal_—*i* element contents in 1 Mg of coal in dry state, mg, *C*_icoke_—*i* element contents in coke obtained from 1 Mg of coal, mg

(2) $$\begin{array}{c} C_{\text{icoal}}=m_{\text{coal}}\cdot {\text{c}}_{\text{icoal}},\\ C_{\text{icoke}}=m_{\text{coke}}\cdot {\text{c}}_{\text{icoke}}, \end{array}$$

where *m*_*i*_ and *c*_*i*_ denote *i* element mass and concentration in coal and coke, respectively.

The calculated values of the release coefficients are shown in Table 5.

The tested charge coal was not only different in the trace elements contents but also in the release coefficient values. Since the pyrolysis conditions were similar, it may be assumed that the release process is affected by other factors such as chemical and mineralogical form, in which trace elements contained in coal are found, and the contents and composition of mineral matter in coal. This, however, should be a subject of separate studies.

### 6.2. Emission of Trace Elements

Trace elements, released in gaseous form in the coal coking process to coke oven gas, such as mercury, or released as chemical compounds, such as the other tested elements, pass into the tar/water condensate and then into the ammonia liquor and products obtained from tar. A small part of the crude coke oven gas escapes into the air as fugitive emission through the leaks in the brickwork of the coke oven, doors and frame leaks, and other technological openings. Due to the fugitive emission, trace elements present in the volatile products of carbonization are carried into the air.

In order to evaluate the fugitive emission of trace elements from the coke oven plant, it is necessary to know the total loss of crude coke oven gas generated in the battery. It may be assumed that the trace elements released from the coked coal pass fully to the gaseous products of carbonization. Thus, if the loss of crude coke oven gas due to leakages is *x*% by volume or mass, the emission of the *i* element released into the air is also *x*% of the released element mass. The loss of crude coke oven gas through the leakages in the battery can only be estimated. Decades ago, the loss of crude coke oven gas was estimated at one to several percent. Owing to technical and technological progress, the loss of gas is now considerably smaller [19].

On the basis of the data on the composition of coke oven gas and the results of the present analyses (Table 4), crude coke oven gas loss *l* has been estimated in % vol. They range from 0.83 to 1.57% vol in Victoria CP, from 1.20 to 2.24% vol in Carbo-Koks CP, from 0.56 do 1.05% vol in Dębieńsko CP, and from 0.21 to 0.40% vol in Przyjaźń CP.

When knowing the contents of the *i* element in 1t of charge coal sample *C*_icoal_ (see (1)), the release coefficient *R*_*i*_ of this element, and the loss *l* of the crude coke oven gas in a particular battery and assuming that the coke yield is 0.76 (information from investigated coke plants), it is possible to estimate the emission factor EF_*if*_ of the *i* element in the production of 1 Mg of coke in the battery

(3) $$\text{E}{\text{F}}_{if}=0.1316\cdot C_{\text{icoal}}\cdot R_{i}\cdot l\cdot 10^{-3}\left[\frac{\text{mg}}{\text{Mg}}\right].$$

The values of fugitive emission factors EF_*if*_ are presented in Table 6.

Knowing the EF_*if*_ values and the annual coke production, it is possible to estimate the *i* element annual fugitive emission to the air, that is, E_*iaf*_ (Table 7)

(4) $${\text{E}}_{iaf}=m_{\text{acoke}}\cdot \text{E}{\text{F}}_{iu}\cdot 10^{-6}\;\left[\text{kg}\right],$$

where, *m*_acoke_—annual coke production in coke oven battery in Mg.

Table 7 presents the ranges of the annual fugitive emission of selected trace elements, calculated in the four coking plants of interest. The data shows that the estimated annual emission of Be, Cd, and Hg does not exceed 1 kg. In the case of the other elements, the emission does not exceed 5 kg for Co, Se, and Tl, 10 kg for As and Ni, and 100 kg for Mn, Sr, V, and Zn. Even if significant, reaching up to 100%, error is assumed, it may be concluded that the emission of the selected trace elements, caused by leaks in the coke oven battery, is lower than previously assumed. Perhaps, the emission was then determined as the amount of the element released in the coking process, and other ways of its penetration to the environment were not considered.

In a few old coking plants, crude coke oven gas is used to fire coke oven batteries. In these cases, trace elements contained in the gas are emitted into the air together with the flue gases through the battery stack, that is, organized emission (emission of pollutants from every kind of technological and combustion processes, released through stationary point sources) takes place. Organized emission is greater than fugitive emission. In the case of old batteries fired with crude coke oven gas, for example, in Carbo-Koks CP, the dominant source of emission is the battery firing stack. Assuming that half of the crude coke oven gas is burnt to heat the coke oven battery, the organized emission factor EF_*io*_ (in reference to the charge coal) is

(5) $$\text{E}{\text{F}}_{io}=0.5\cdot C_{\text{icoal}}\cdot R_{i}\;\left[\frac{\text{mg}}{\text{Mg}}\right],$$

where, *C*_icoal_ contents of *i* component in 1 Mg of coal in dry state, mg, *R*_*i*_ release coefficient of *i* element

Annual organized emission *E*_*io*_ may be estimated after the following equation:

(6) $${\text{E}}_{io}=m_{\text{acoal}}\cdot \text{E}{\text{F}}_{io}\cdot 10^{-6}\;\left[\text{kg}\right],$$

where, *m*_acoal_ annual consumption of charge coal, Mg.

Table 8 shows the emission factors of the selected trace elements found for firing the battery in Carbo-Koks CP.

The calculated value of the organized emission from the battery firing is several times higher than the value of the fugitive emission resulting from the coke gas loss. The relevant calculation data acquired for Carbo-Koks CP is compared in Table 9.

## 7. Summary

The present research is an initial stage and a first step to evaluate the emission of trace elements from a coke ovens into the air. The results obtained refer to four coke oven batteries and properties of the charge coal used in the tests. The calculated values of the release coefficients, emission factors, and annual emission are approximate. If the technical and technological conditions in the coking plants can be considered stable, the contents of trace elements in the charge coal vary widely. Owing to highly different contents of trace elements in coals and various chemical and mineral forms in which they occur, reliable assessment of the air emission from coking processes requires a larger study. The selection of samples should take into account the share weighting of individual coking plants in the national coke production. Another issue is the knowledge of the distribution of trace elements in other, different from coke, products of coal coking.

The present studies have shown that although the results refer to the tested cases, it may be concluded that the emission of the tested trace elements from the modern coke oven battery into the air is relatively small and does not constitute a greater threat to the environment. Further studies should verify this view.

## 8. Conclusions

- In result of analyses of charge coal and coke obtained from it, the release coefficients were determined in the process of charge coal coking in four coke oven batteries for arsenic, beryllium, cobalt, cadmium, mercury, manganese, nickel, selenium, strontium, thallium, vanadium, and zinc.
- Concentrations of individual elements in charge coal samples vary considerably. The lowest values, from a few to more than 100 ppb, are for Cd and Hg. The highest values, exceeding 100 ppm and 200 ppm, are Mn and Sr concentrations, respectively.
- The release coefficients of trace elements are varied, and the range of their variability is from 0.5 (strontium) to 94% (mercury).
- On the basis of the data on the content of trace elements in charge coals, release coefficients, and crude coke oven gas loss, is possible to estimate the emission factor of tested element in the production of 1 Mg of coke in the battery and knowing battery capacity annual fugitive emission of tested elements.
- Fugitive emission of the trace elements investigated, occurring due to coke oven leaks and openings, is small and is not a real threat to the environment.
- Basing on the release coefficients values, emission factors of the selected trace elements were calculated in the process of battery firing with crude coke oven gas. It can be stated that the calculated, using battery capacity, value of the organized emission from the battery firing is several times higher than the value of the fugitive emission resulting from the coke gas loss.

## Figures and Tables

**Table 1:** The optimum measurement conditions.

Rf power [W]	1025
Plasma gas flow [L/min]	15
Nebulizer gas flow [L/min]	0.7–0.76
Auxiliary gas flow [L/min]	1.13
Nebulizer	Cross flow
Plasma torch	quartz
Sample uptake [mL/min]	1
Scanning mode	Peak hop
Dwell time [ms]	100
Sweeps/reading	20
Number of replicates	3
Read delay time [s]	15
Cell gas	CH_4_
DRC gas flow	0.7
RPq (rejection parameter q)	0.65

**Table 2:** The detection and quantification limits of analyzed elements.

Isotope	Limit of	Limit of
	detection (LOD)	quantification (LOQ)
	[*μ*g/L]	[*μ*g/L]
^75^As	0.20	0.60
^9^Be	0.09	0.27
^114^Cd	0.03	0.09
^59^Co	0.01	0.03
^78^Se	0.02	0.06
^88^Sr	0.02	0.06
^205^Tl	0.03	0.09
^55^Mn	0.03	0.09
^98^Mo	0.01	0.03
^60^Ni	0.05	0.15
^66^Zn	0.18	0.54
^65^Cu	0.06	0.18
^51^V	0.03	0.09

**Table 3:** Results of the technical and elemental analysis of the tested charge coal and coke and uncertainty of the results.

No.	Coking plant	Kind of sample	Contents in the analytical sample (% weight)						
			Volatile matter	Moisture	Ash	S	C	H	N
			daf	ad	ad	daf			
(1)	Victoria	Charge coal	22.05 ± 0.35	1.26 ± 0.04	7.62 ± 0.08	0.46 ± 0.05	83.91 ± 0.23	4.55 ± 0.23	0.97 ± 0.23
		Coke		1.60 ± 0.20	11.09 ± 0.17	0.66 ± 0.02	86.85 ± 0.23	1.11 ± 0.23	0.62 ± 0.23
(2)	Carbo-Koks	Charge coal	24.71 ± 0.35	1.58 ± 0.04	8.02 ± 0.08	0.60 ± 0.05	82.07 ± 0.23	4.48 ± 0.23	0.92 ± 0.23
		Coke		1.50 ± 0.20	11.65 ± 0.17	0.52 ± 0.02	86.96 ± 0.23	0.60 ± 0.23	0.75 ± 0.23
(3)	Dębieńsko	Charge coal	30.07 ± 0.35	2.04 ± 0.04	5.86 ± 0.08	0.37 ± 0.05	82.33 ± 0.23	5.00 ± 0.23	1.01 ± 0.23
		Coke		1.10 ± 0.20	9.54 ± 0.17	0.36 ± 0.02	91.40 ± 0.23	0.58 ± 0.23	0.84 ± 0.23
(4)	Przyjaźń	Charge coal	23.53 ± 0.35	1.28 ± 0.04	7.64 ± 0.08	0.56 ± 0.05	84.12 ± 0.23	4.90 ± 0.23	0.94 ± 0.23
		Coke		0.70 ± 0.20	10.52 ± 0.17	0.52 ± 0.02	90.73 ± 0.23	0.52 ± 0.23	0.72 ± 0.23

**Table 4:** Concentration of the selected trace elements in the charge coal and coke obtained.

No.	Coking plant	Kind of sample	Concentration of the selected elements *N* = 9 (ppb)											
			As	Be	Cd	Co	Hg	Mn	Ni	Se	Sr	Tl	V	Zn
(1)	Victoria	Charge coal	2,43·10^3^	1,05·10^3^	4,20·10^1^	6,82·10^3^	7,80·10^1^	9,75·10^4^	2,12·10^4^	5,61·10^2^	7,80·10^4^	1,80·10^2^	5,08·10^4^	4,25·10^4^
		Coke	2,98·10^3^	1,29·10^3^	<LOD	8,47·10^3^	1,90·10^1^	1,20·10^5^	2,59·10^4^	6,00·10^2^	9,20·10^4^	1,71·10^2^	5,90·10^4^	5,39·10^4^
(2)	Carbo-Koks	Charge coal	3,04·10^3^	6,58·10^2^	1,43·10^2^	4,29·10^3^	6,70·10^1^	4,24·10^4^	1,10·10^4^	9,59·10^2^	9,11·10^4^	6,99·10^2^	3,12·10^4^	4,22·10^4^
		Coke	2,07·10^3^	8,33·10^2^	5,60·10^1^	5,51·10^3^	9,00	5,55·10^4^	1,32·10^4^	7,87·10^2^	1,20·10^5^	2,89·10^2^	3,85·10^4^	5,26·10^4^
(3)	Dębieńsko	Charge coal	5,43·10^2^	<LOD	2,40·10^1^	<LOD	8,70·10^1^	5,30·10^4^	1,05·10^4^	3,19·10^2^	1,69·10^5^	2,06·10^2^	3,89·10^4^	2,18·10^4^
		Coke	7,46·10^2^	<LOD	<LOD	<LOD	7,00	7,45·10^4^	1,42·10^4^	3,88·10^2^	2,27·10^5^	1,52·10^2^	4,48·10^4^	2,16·10^4^
(4)	Przyjaźń	Charge coal	2,46·10^3^	8,19·10^2^	1,19·10^2^	5,96·10^3^	7,00·10^1^	5,69·10^4^	1,40·10^4^	8,98·10^2^	1,05·10^5^	2,20·10^2^	4,56·10^4^	3,60·10^4^
		Coke	2,80·10^3^	7,67·10^2^	4,50·10^1^	6,19·10^3^	2,10·10^1^	6,82·10^4^	1,58·10^4^	1,09·10^3^	1,36·10^5^	2,84·10^2^	3,68·10^4^	2,99·10^4^

<LOD: below limit of detection.

*N*: number of measurements.

**Table 5:** Release coefficients of the selected trace elements.

No.	Coking plant	Release coefficients of the selected trace elements (%)											
		*R*_As_	*R*_Be_	*R*_Cd_	*R*_Co_	*R*_Hg_	*R*_Mn_	*R*_Ni_	*R*_Se_	*R*_Sr_	*R*_Tl_	*R*_V_	*R*_Zn_
(1)	Victoria	3.96	3.80	n.d.a.	2.64	80.72	3.33	3.89	15.99	7.56	25.17	8.88	0.48
(2)	Carbo-Koks	48.45	3.97	70.56	2.50	89.93	0.70	9.04	37.78	0.42	68.68	6.44	5.50
(3)	Dębieńsko	2.79	n.d.a.	n.d.a.	n.d.a.	93.98	0.51	4.02	13.96	5.22	47.86	18.50	30.09
(4)	Przyjaźń	12.39	28.09	70.79	20.27	76.62	7.86	13.48	7.12	0.66	0.97	37.98	36.05

n.d.a.: no data available.

**Table 6:** Ranges of fugitive emission factors EF_*if*_ of the selected trace elements.

Coking plant	Fugitive emission factor EF*_if_* [mg/Mg]											
	As	Be	Co	Cd	Hg	Mn	Ni	Se	Sr	Tl	V	Zn
Victoria	1.1–2.0	0.4–0.8	2.0–3.7	n.d.a.	0.7–1.3	35.5–67.1	9.0–17.0	1.0–1.9	64.4–121.9	0.5–0.9	49.3–93.2	2.2–4.2
Carbo-Koks	23.3–43.4	0.4–0.8	1.7–3.2	1.6–3.0	0.9–1.8	4.7–8.7	15.7–29.4	5.7–10.7	6.0–11.3	7.6–14.1	31.7–59.2	36.6–68.4
Dębieńsko	0.1-0.2	n.d.a.	n.d.a.	n.d.a.	0.6–1.1	2.0–3.7	3.1–5.8	0.3–0.6	65.2–122.2	0.7–1.4	53.1–99.5	48.4–90.8
Przyjaźń	0.8–1.6	0.6–1.2	3.3–6.4	0.2–0.4	0.1–0.3	12.4–23.5	5.2–9.9	0.2–0.3	1.9–3.7	0.006–0.011	47.9–91.2	35.8–68.2

n.d.a.: no data available.

**Table 7:** Ranges of estimated annual fugitive emission of trace elements from the tested coke oven.

Coking plant	Estimated annual fugitive emission of the element [kg]											
	As	Be	Cd	Co	Hg	Mn	Ni	Se	Sr	Tl
Victoria	0.5–1.0	0.2–0.4	n.d.a.	1.0–1.9	0.3–0.7	17.7–33.5	4.5–8.5	0.5–0.9	32.2–61.0	0.2–0.5	24.6–46.6	1.1–2.1
Carbo-Koks	3.0–5.6	0.1	0.2–0.4	0.2–0.4	0.1–0.2	0.6–1.1	2.0–3.8	0.7–1.4	0.8–1.5	1.0–1.8	4.1–7.7	4.8–8.9
Dębieńsko	0*–0.1	n.d.a.	n.d.a.	n.d.a.	0.2–0.3	0.5–0.9	0.8–1.5	0.1–0.2	16.3–30.6	0.2–0.3	13.3–24.9	12.1–22.7
Przyjaźń	0.6–1.2	0.5–0.9	0.2–0.3	2.5–4.8	0.1–0.2	9.3–17.6	3.9–7.4	0.1–0.3	1.4–2.7	0*	35.9–68.4	26.9–51.2

n.d.a.: no data available.

*<0.1 kg.

**Table 8:** Organized emission factors EF_*io*_ of selected trace elements found for firing the coke oven battery in Carbo-Koks CP, mg/Mg.

Trace element	EF*_io_*
As	737
Be	13
Co	54
Cd	50
Hg	30
Mn	148
Ni	496
Se	181
Sr	191
Tl	240
V	1004
Zn	1159

**Table 9:** Annual emission from the coke oven battery in Carbo-Koks CP.

Emission	Annual emission of the element [kg]											
	As	Be	Cd	Co	Hg	Mn	Ni	Se	Sr	Tl	As	Zn
Fugitive	3.0–5.6	0.1	0.2–0.4	0.2–0.4	0.1–0.2	0.6–1.1	2.0–3.8	0.7–1.4	0.8–1.5	1.0–1.8	4.1–7.7	4.8–8.9
Organized	96.9	6.6	2.7	7.0	3.9	19.5	65.5	23.8	25.1	31.5	132.0	152.5

## References

[1] U.S. EPA Study of hazardous air pollutant emissions from electric utility steam generating units–Final Report to Congress.

[2] Jakowlewa TP, Doujanskaja JB (1995). Toxic trace elements in wastes released to environment during coal pyrolysis. *Coke and Chemistry*.

[3] Zajusz-Zubek E, Konieczyński J (2003). Dynamics of trace elements release in a coal pyrolysis process. *Fuel*.

[4] Li Y, Zhang JY, Zhao YC, Wu YQ, Gao JS, Zheng CG (2007). Influence of pyrolysis conditions on volatility of trace elements in coals. *Journal of Engineering Thermophysics*.

[5] Li Y, Zhang J, Wu Y, Zheng C (2008). Volatility of trace elements in the coal from Shenfu during pyrolysis. *Journal of Huazhong University of Science and Technology (Natural Science Edition)*.

[6] Yiwei C, Guijian L, Yanming G, Jianli Y, Cuicui Q, Lianfei G (2007). Release and enrichment of 44 elements during coal pyrolysis of Yima coal, China. *Journal of Analytical and Applied Pyrolysis*.

[7] Chen Y, Liu G, Wang L, Kang Y, Yang J (2008). Occurrence and fate of some trace elements during pyrolysis of Yima coal, China. *Energy and Fuels*.

[8] Liu R, Yang J, Xiao Y, Liu Z (2009). Fate of Forms of arsenic in Yima coal during pyrolysis. *Energy and Fuels*.

[9] Boyd RJ (2004). *Trace elements in coal from Collinsville, Bowen basin, Australia–in-ground mode of occurrrence and behaviour during utilization*.

[10] Eisenhut W (1991). *DMT - Gesellschaft für Forschung und Prüfung *.

[11] Pacyna JM, Pacyna EG (2001). An assessment of global and regional emissions of trace metals to the atmosphere from anthropogenic sources worldwide. *Environmental Reviews*.

[12] Emission Inventory Guidebook.

[13] Pacyna Combustion and Industry Expert Panel Workshop.

[14] EMEP/EEA Emission Inventory Guidebook.

[15] Theloke J, Kummer U, Nitter S, Geftler T, Friedrich R Ueberarbeitung der Schwermetallkapitel im Corinair Guidebook zur Verbesserung der Emissioninventare und der Berichterstattung im Rahmem der Genfer Luftreinhaltekonvention.

[16] Roy LC, Chakrovarty DK (1984). Impact of trace elements of coal utilization. *Chemical Economy & Engineering Review*.

[17] Dębska-Bes M, Menzla H (1989). Toxic elements in coking coals and in coking industry issues. *Koks, Smoła, Gaz*.

[18] Menzla H, Dębska-Bes M (1990). Concentration of toxic elements in feed stream and products of plants for mechanical processing of energy and coking coals. *Koks, Smoła, Gaz*.

[19] Zieliński H (1986). *Koksownictwo*.

